# Organizational readiness to change as a leverage point for improving safety: a national nursing home survey

**DOI:** 10.1186/s12913-021-06772-y

**Published:** 2021-08-20

**Authors:** Emma D. Quach, Lewis E. Kazis, Shibei Zhao, Pengsheng Ni, Valerie A. Clark, Sarah E. McDannold, Christine W. Hartmann

**Affiliations:** 1 Center for Healthcare Organization and Implementation Research, VA Bedford Healthcare System (152), 200 Springs Road, Bedford, MA 01730 USA; 2 New England Geriatric Research Education and Clinical Center, VA Bedford Healthcare System, Bedford, USA; 3 Department of Gerontology, McCormack Graduate School of Policy and Global Studies, University of Massachusetts Boston, 100 Morrissey Boulevard, Boston, MA 01225 USA; 4 Health Outcomes Unit and Center for the Assessment of Pharmaceutical Practices (CAPP), Department of Health Law, Policy and Management, Boston University School of Public Health, 715 Albany Street, Talbot 5 West (532), Boston, MA 02118 USA; 5 Spaulding Outcomes Rehabilitation Center, Spaulding Hospital, Charlestown, MA USA; 6 Visiting Professor, Harvard Medical School and Research Director Spaulding Outcomes Rehabilitation Center, Spaulding Hospital, Charlestown, MA USA; 7 Health Law, Policy & Management, Department of Health Law, Policy and Management, Boston University School of Public Health, 715 Albany Street, Talbot 3 West (532), Boston, MA 02118 USA; 8 Department of Public Health, Zuckerberg College of Health Sciences, University of Massachusetts Lowell, 61 Wilder St., O’Leary 540, Lowell, MA 01854 USA

**Keywords:** Safety culture, Safety climate, Safety, Organizational readiness to change, Organizational change, Surveys, Nursing homes, Long term care

## Abstract

**Background:**

A stronger safety climate in nursing homes may reduce avoidable adverse events. Yet efforts to strengthen safety climate may fail if nursing homes are not ready to change. To inform improvement efforts, we examined the link between organizational readiness to change and safety climate.

**Methods:**

Seven safety climate domains and organizational readiness to change were measured with validated Community Living Center/CLC Employee Survey of Attitudes about Resident Safety and Organizational Readiness to Change Assessment. Safety climate domains comprised of safety priorities, supervisor commitment to safety, senior management commitment to safety, safety attitudes, environmental safety, coworker interactions around safety, and global rating of CLC. We specified models with and without readiness to change to explain CLC- and person-level variance in safety climate domains.

**Results:**

One thousand three hundred ninety seven workers (frontline staff and managers) responded from 56 US Veterans Health Administration CLCs located throughout the US. Adding readiness to change reduced baseline CLC-level variance of outcomes (2.3–9.3%) by > 70% for interpersonal domains (co-workers, supervisors, and senior management). Readiness to change explained person-level variance of every safety climate domain (*P* < 0.05), especially for interpersonal domains.

**Conclusions:**

Organizational readiness to change predicted safety climate. Safety climate initiatives that address readiness to change among frontline staff and managers may be more likely to succeed and eventually increase resident safety.

## Background

In nursing homes, avoidable safety-related adverse events occur fairly consistently, despite improvement efforts [1] and result in resident morbidity and mortality [2]. Adverse events or actual harm were reported by more than one-fifth of individuals admitted to nursing homes, with over half of these adverse events avoidable [3]. Among the most common, and publicly reported, avoidable adverse events in nursing homes are pressure ulcers; health care-related infections; and fracture and head trauma [1]. Nursing homes struggle to prevent avoidable adverse events, however this is not well understood [1].

Evidence, sometimes equivocal [4] increasingly supports the potential of safety climate to enhance patient safety outcomes. Safety climate is defined as safety-related behavioral and attitudinal norms among organizational members and is measured by multiple domains: members’ perceptions about safety priorities and commitment by supervisors and senior management to safety [5]. Safety climate interventions may thus hold promise to prevent adverse events, with successful interventions in hospitals citing organizational context as a contributor to their success [6, 7]. Successful hospital interventions have encompassed multiple components, such as team training and executive walk rounds (during which managers engage with frontline staff in discussions about safety), with success contingent upon organizational contextual factors such as the size and staffing (frontline and managerial) of the organization and the infrastructure for staff communications [6, 8]. Yet safety climate initiatives in nursing homes are sparse [6], possibly because nursing homes may lack readiness to change [9].

Organizational readiness to change refers to an organization’s psychological and behavioral readiness for general change. Psychological readiness comprises openness to change among frontline staff and managers and behavioral readiness to system resources, with readiness linked to adoption of change [10]. Yet, when studied in hospitals in the United Kingdom and Sweden, organizational readiness to change had mixed associations (positive and null) with safety climate [11, 12]. Findings in hospitals may not generalize to nursing homes because of important personnel differences, creating a need to study organizational readiness and safety climate in the nursing home context. For example, most of the direct care in nursing homes, unlike in hospitals, is performed by certified nursing assistants and licensed practical nurses [13]. Direct care work is performed under high work pressure, with high turnover among direct care workers [14]. A changing cadre of workers occupied with resident care may pose a challenge to nursing home administrators seeking to inform staff of new change initiatives and obtain their buy-in. Such context may thus make organizational readiness to change in nursing homes difficult to achieve but highly salient to efforts to change safety climate.

We thus sought to inform the implementation of safety climate interventions in nursing homes by examining the association of organizational readiness to change with safety climate. Our sample of nursing homes consists of US Department of Veterans Affairs (VA) Community Living Centers (CLCs) affiliated with VA medical centers, operating alongside US non-profit and for-profit nursing homes. Although the average CLC differs from the average nursing home in the US [15] because it currently serves mostly male and relatively younger residents, all CLCs are similar to other US nursing homes because they must adhere to resident care standards, undergo external inspections, address deficiencies, and publicly report adverse events [16]. Our investigation of VA CLCs may offer particular insights for nursing homes affiliated with hospitals, i.e., in integrated health systems.

## Methods

### Study design

The research design comprised of a cross-sectional survey of all VA nursing homes in the US (132) using 2 validated survey instruments administered to nursing home employees, the CLC Employee Survey of Attitudes about Resident Safety [17] and the Organizational Readiness to Change Assessment [18].

### Data collection

We used the previously validated CLC Employee Survey of Attitudes about Resident Safety (CESARS) [17] to assess 7 safety climate domains: safety priorities (prioritization of safety processes), supervisor commitment to safety (e.g., supervisor’s availability to discuss resident safety), senior management commitment to safety (e.g., senior managers’ knowledge about resident safety), safety attitudes (e.g., feeling responsible for resident safety issues), environmental safety (physical hazards), coworker interactions around safety (e.g., teamwork around safety), and global rating of CLC (e.g., willingness to recommend CLC). The CESARS also asked for respondents’ occupation, CLC tenure, work shift, and weekly hours.

We assessed organizational readiness to change with the validated Organizational Readiness to Change Assessment (ORCA)‘s Organizational Context for Change domain [18]. This domain comprises sub-domains related to psychological readiness (openness to change among senior management, opinion leaders, and staff) and to behavioral readiness (staff empowerment by senior management, information gathering and employee feedback, and allocating system resources for change). For clarity, we chose to call this domain Psychological and Behavioral Readiness to Change.

Between April 2016 and May 2017, we contacted all 132 VA CLCs (the entire population of VA CLCs). Fifty-six (42%) gave us staff lists. The CESARS and ORCA were anonymously administered either online or by mail to the 56 CLCs. Additional data about structure and process of care were obtained from the VA Geriatrics and Extended Care Data Analytics Center, VA Office of Productivity, Efficiency, and Staffing, and the VA Field Research Advisory Committee. In addition, the VA All-Employee Survey, an anonymous annual survey of job satisfaction/attitudes for all active VA employees (330,732 invited, with a 60% response rate), was merged with these data [19].

.Approval for this study was obtained from the VA Bedford Healthcare System institutional review board, which granted a waiver of written informed consent.

### Variables

The CESARS 7 safety climate domains constitute seven person-level outcome variables. Each of these domains was scaled by standardizing them to a mean of 50 and a standard deviation of 10 using the sample of CLC’s that responded to the survey. The internal consistency reliability of the 7 scales ranged from 0.60–0.96.

The primary independent variable was the CLC-level mean of the Psychological and Behavioral Readiness to Change domain ranging from 1 to 5, denoting less to more readiness. We computed the CLC-level Psychological and Behavior Readiness to Change as the mean of responses of all staff members in the same CLC regardless of job type after observing that responses within the same CLC did not significantly differ by job type.

Four person-level covariates were occupation (nursing assistants, licensed nurses, clinicians/specialists, senior managers, and administrative/support staff); CLC tenure in years (< 2, 2 < 5, ≥ 5 < 10, and ≥ 10); shift (day/night); and usual work hours per week (≤ 40 versus > 40).

We created five CLC-level covariates. Four were created by aggregating characteristics of each CLC’s employees: % of a CLC’s respondents who 1) were licensed nurses, 2) had CLC tenure ≥5 years, 3) worked day shift, and 4) worked ≤40 h/week. Such aggregation enabled us to address whether staffing homogeneity (e.g., co-workers with same occupation) influenced safety climate [20]. A fifth variable, CLC’s nonresponse rate to the CESARS, captured unwillingness to complete a survey about one’s CLC’s safety climate.

Five other CLC-level covariates were related to the structure and process of care: CLC size (FY2017 operating beds), nursing ratio (FY2017 nursing hours per bed per day), complexity level of the CLC’s affiliated VA hospital (low/medium/high), geographic location (Mid-Atlantic, Midwest, Northeast, South, or West), and job satisfaction/engagement of employees in the CLC (1–5 range, lower to higher satisfaction/engagement).

### Analysis

We assessed the influence of Psychological and Behavioral Readiness to Change on the CLC-level and the person-level variance of safety climate domain. We performed 4 sequential multivariate regression models for each safety climate domain. The baseline models (Step 1) contained only person-level occupation, job tenure, work shift, and work hours. Subsequent models and their respective independent variables were as follows: Steps 2) eight CLC-level variables (all except job satisfaction/engagement, nursing ratio, and Psychological and Behavioral Readiness to Change), 3) job satisfaction/engagement plus significant variables (*P* < 0.05) from Step 2, and 4) nursing ratio and Psychological and Behavioral Readiness to Change plus significant variables from Step 3. We examined the difference between the baseline and each subsequent model’s intraclass correlations (ICCs) [21] to check for reductions in ICC values, as such reductions indicate variables explaining CLC-level variance in safety climate; we further examined regression coefficients in step 4 for independent associations between variables and person-level variance in safety climate. Analysis used SAS version 9.3 SAS Institute, Inc., Cary NC.

## Results

Table 1 describes 1397 respondents (26%) and 56 CLCs. Respondents included licensed nurses (52%), clinicians (e.g., physicians, 14%), and senior management (e.g., medical directors, 6%). Psychological and Behavioral Readiness to Change averaged at 3.28 points (1–5).

**Table 1:** Characteristics of CLCs and their staff respondents

Variable	N (%) or mean ± standard deviation
Person-level characteristics (*n* = 1397)	
Occupation type	
Nursing assistants	333 (24.4)
Licensed nurses	711 (52.1)
Clinicians/specialists	192 (14.1)
Senior managers	80 (5.9)
Administrative/support	49 (3.6)
CLC tenure in years	
< 2 years	445 (32.6)
≥ 2 years < 5 years	340 (24.9)
≥ 5 years < 10 years	310 (22.7)
≥ 10 years	272 (19.9)
Work shift	
Day	673 (49.4)
Night, evening and others	690 (50.6)
Work hours per week	
≤ 40 h	974 (70.7)
> 40 h	403 (29.3)
CLC-level characteristics (*n* = 56)	
Occupation: licensed nurses (%)	53.9 ± 17.2
Tenure: ≥ 5 years (%)	46.2 ± 16.2
Work shift: day shift (%)	51.7 ± 18.3
Work hours: ≤ 40 h/week (%)	72.0 ± 13.3
Non-response rate	4.4 ± 2.6
Number of operating beds	100.3 ± 60.7
Nursing ratio	6.8 ± 1.5
VA hospital complexity	
Low	13 (23.2)
Medium	24 (42.9)
High	19 (33.9)
CLC location	
Mid-Atlantic	12 (21.4)
Midwest	11 (19.6)
Northeast	9 (16.1)
South	14 (25.0)
West	10 (17.9)
Job satisfaction/ engagement^a^	3.80 ± 0.21
Psychological and behavioral readiness to change^a^	3.28 ± 0.34

*Note*: *CLC* Community Living Centers. Totals may not add to 100 due to rounding or missing item-level data. ^a^ Range for job satisfaction/engagement and Psychological and Behavioral Readiness to Change is 1–5

Table 2 shows ICCs from each step and differences between Step 1 and each subsequent step. With only person-level covariates, ICCs ranged between 2.3 to 9.3%, with particularly high ICC’s for supervisor and senior management commitment to safety (higher values indicate higher similarity among employees in the same CLC and a need for CLC-level variables). ICC comparisons indicated that the staffing ratio and Psychological and Behavioral Readiness to Change domain explained more CLC-level variance in the safety climate domains than any other variable. In particular, for supervisor commitment, the ICC dropped by 18.5% between Step 1 and 3 but by 72% between Step 1 and 4; similar reductions were also observed for coworker interactions and for senior management commitment to safety (5% between Step 1 and 3 but 77% between Step 1 and 4).

**Table 2:** Intraclass coefficients from Steps 1–4 random effects regressions for each safety climate domain outcome

Safety climate domain	Step 1 ICC (SE)	Step 2 ICC (SE)	Step 3 ICC (SE)	Step 4 ICC (SE)	Absolute and (%) of ICC change between Step 1 and Step 2	Absolute and (%) of ICC change between Step 1 and Step 3	Absolute and (%) of ICC change between Step 1 and Step 4
Safety priorities	0.028* (0.012)	0.019 (0.012)	0.013 (0.009)	0.003 (0.008)	0.009 (− 29.8%)	0.015 (− 53.8%)	0.025 (− 90.0%)
Supervisor commitment	0.099*** (0.023)	0.080*** (0.023)	0.083*** (0.021)	0.027* (0.013)	0.019 (− 18.5%)	0.016 (− 15.8%)	0.072 (− 72.2%)
Senior management commitment	0.093*** (0.024)	0.093 *** (0.028)	0.089*** (0.023)	0.021 (0.011)	0 (− 0.4%)	0.004 (− 4.8%)	0.072 (− 77.6%)
Attitudes towards safety	0.023* (0.012)	0.025 (0.014)	0.023 (0.012)	0.016 (0.011)	0.002 (6.7%)	0 (− 3.9%)	0.007 (− 30.4%)
Environmental safety	0.093*** (0.024)	0.102** (0.030)	0.078*** (0.021)	0.070*** (0.020)	0.009 (9.6%)	0.015 (− 16.4%)	0.023 (− 24.6%)
Co-worker interactions	0.064*** (0.019)	0.072** (0.023)	0.059** (0.018)	0.009 (0.009)	0.008 (13.4%)	0.005 (− 7.2%)	0.055 (− 85.3%)
Global ratings	0.094*** (0.023)	0.112*** (0.03)	0.075*** (0.018)	0.053** (0.017)	0.018 (19.6%)	0.019 (− 19.6%)	0.041 (− 29.7%)

Each model was 2-level linear mixed effects model with a random intercept for clustering by CLC. Step 1 included only person-level covariates (occupation, CLC tenure, work shift, and weekly hours). Step 2 included all person-level covariates and all CLC-level covariates except employee satisfaction/engagement, nursing ratio, and Psychological and Behavioral Readiness to Change. Step 3 included all Step 2’s significant covariates and job satisfaction/engagement. Step 4 contained all Step 3’s significant covariates and nursing ratio and Psychological and Behavioral Readiness to Change

*ICC* Intraclass correlations, *SE* Standard error

* *P* < .05; ** *P* < .01; *** *P* < .001

Step 4 variables--nursing ratio and Psychological and Behavioral Readiness to Change—made substantial contributions to explaining differences across CLCs in terms of safety priorities, supervisor commitment, senior management commitment, and co-worker interactions

Table 3 shows Step 4 models, with Psychological and Behavioral Readiness to Change significantly associated with all 7 safety climate domains. The strongest associations were observed with supervisor, co-worker, and senior management domains of safety climate. For example, one point in Psychological and Behavioral Readiness to Change was associated with nearly 0.80 of one standard deviation in senior management commitment to safety.

**Table 3:** Safety climate domain outcomes regressed on Psychological and Behavioral Readiness to Change Domain, controlling for person- and CLC-level characteristics

	Safety climate domains						
	Safety priorities	Supervisor commitment to safety	Senior management commitment to safety	Attitudes towards safety	Environmental safety	Co-worker interactions around safety	Global rating of the CLC
	*B* (SE)	*B* (SE)	*B* (SE)	*B* (SE)	*B* (SE)	*B* (SE)	*B* (SE)
**Person-level variables**							
Occupation type							
Nursing assistants	1.72*** (0.46)		3.64*** (0.58)	−0.38 (0.48)		0.52 (0.51)	1.46* (0.58)
Clinicians/specialists	−0.52 (0.56)		3.00***	−3.61***(0.60)		1.34* (0.63)	0.99 (0.74)
Administrative/support	0.13 (1.03)		3.47** (1.32)	−2.99** (1.07)		2.92* (1.13)	2.85* (1.32)
Senior managers	1.15 (0.81)		6.02*** (1.03)	2.09* (0.85)		4.17*** (0.90)	5.14*** (1.03)
Licensed nurses	Ref.	Ref.	Ref.	Ref.	Ref.	Ref.	Ref.
CLC Tenure							
< 2 years			1.73* (0.66)	3.03*** (0.55)			−0.15 (0.68)
≥ 2 years- < 5 years			−0.58 (0.69)	−1.78** (0.58)			−1.82* (0.71)
≥ 5 years- < 10 years			−1.69* (0.71)	− 1.27* (0.60)			−0.68 (0.73)
≥ 10 years	Ref.	Ref.	Ref.	Ref.	Ref.	Ref.	Ref.
Work shift							
Day/Night		1.0* (0.48)	1.84*** (0.51)				
Work hours per week							
≤ 40 versus > 40 h		1.01 (0.53)					
**CLC-level variables**							
Occupation: licensed nurses (%)							
Tenure: ≥ 5 years (%)	4.14** (1.52)	7.57** (2.36)					
Work shift: day shift (%)							
Work hours: ≤ 40 h/week (%)							
CLC size							
Job satisfaction/ engagement	1.52 (1.09)				4.62* (2.07)	0.42 (1.29)	3.70 (1.97)
Nursing ratio	−0.01 (0.14)	−0.12 (0.22)	−0.19 (0.21)	0.18 (0.17)	0.43 (0.28)	0.01 (0.17)	0.25 (0.26)
Psychological and Behavioral Readiness to Change	2.77*** (0.65)	7.46*** (1.01)	7.86*** (0.93)	2.12** (0.75)	2.51 (1.30)	6.15*** (0.79)	5.03*** (1.23)

Note: Variables specified in each Step 4 model were based on Steps 1–3 models as follows. Step 1 models contained only person-level covariates. Step 2 models contained all person-level covariates and all CLC-level variables except job satisfaction/engagement, nursing ratio, and Psychological and Behavioral Readiness to Change Domain. Step 3 included all Step 2’s significant covariates and job satisfaction/engagement. Step 4 contained all Step 3’s significant covariates and nursing ratio and Psychological and Behavioral Readiness to Change Domain. All outcome variables (safety climate domains) were standardized to a mean of 50 and standard deviation of 10. Range for job satisfaction/engagement and Psychological and Behavioral Readiness to Change Domain was 1–5

* *P* < .05; ** *P* < .01; *** *P* < .001; B regression coefficient, *SE* Standard error

Psychological and Behavioral Readiness to Change was consistently and strongly associated with all safety climate domains in CLCs

## Discussion

Across CLCs nationwide, we found organizational readiness to change was related to seven safety climate domains, especially domains related to co-workers, supervisors, and senior managers. These associations highlight the conceptual framework that readiness to change at multiple organizational levels (among peers, between supervisor and supervisee, and among managers) is a requisite aspect for safety climate improvements [22]. Thus, readiness dimensions--leadership and frontline staff openness and information exchange between them—may lay the groundwork for safety climate interventions to succeed and eventually prevent adverse events.

Our findings add to existing evidence that readiness to change in nursing homes, demonstrated by openness to change in frontline staff, opinion leaders, and senior managers and by communications between them, is an essential ingredient in a strong safety climate. Organizational readiness to change may thus be a potential leverage point that safety climate initiatives may address [22]. The association between nursing home readiness to change and safety climate helps explain the inconsistent outcomes of safety rounds by senior managers, an initiative wherein senior managers engage with frontline staff to learn from them and collaboratively resolve safety issues [8]. Safety rounds may have had inconsistent success in some hospitals or nursing homes because staff were not primed for change, e.g., they were not aware a problem in safety climate existed or did not understand the objectives or context for the safety round initiative [23]. Safety rounds’ success will also be compromised if senior managers are not ready for change, do not embrace the change initiative, and go through the motions without seriousness [24].

Safety climate initiatives in nursing homes may have greater success if they incorporate key readiness elements, based on evidence from prior initiatives to enhance organizational readiness to change. Safety climate initiatives need to make it clear to staff of the problem at hand and how the proposed initiative is an appropriate solution to the problem [23]. Because frontline staff may have concerns about the burden to implement the solution, they may be more open to the solution if it does not increase their already high workload [25]. Safety climate initiatives also need to incorporate sustained, hands-on supervisor and senior management engagement and support for the proposed change [25, 26].

### Study limitations

Our findings should be interpreted with caution. Cross-sectional data show an association, not causality, between readiness to change and safety climate. The study design, including a sample of only VA nursing homes, limits generalizability to only VA nursing homes. We do note that our sample was diverse in VA service network and occupations. We also compared nursing homes in our study sample with those who did not respond to our survey using data available on all VA nursing homes. We found no significant differences in geographic region and facility complexity (based on a facility’s patient volume; risk; and clinical, teaching, and research capability) between non-responding nursing homes and our nursing home sample.

### Practice and research implications

The strong associations between organizational readiness to change and safety climate in nursing homes have the following implications for practice and research. Safety climate interventions should first assess and address staff and system readiness to change. Readiness to change assessments and safety climate interventions may also need repeating [23] as staff turnover brings in new staff and may change these dynamics. Whether staff skills and knowledge moderate the association of readiness to change and safety climate should also be examined in future research. Staff members willing to change need adequate skills/knowledge to make safety-related change. For example, senior managers making walk rounds may need training in active listening for these rounds to be effective while new direct care personnel may lack knowledge of warning signs related to resident safety. This research can inform whether organizational readiness to change should include an assessment of skills/knowledge requisite for a particular safety climate intervention, laying the groundwork for future safety climate interventions to succeed.

## Conclusions

Organizational readiness to change predicted safety climate domains in VA nursing homes in the US. This pertained to those domains involving staff interactions to promote safety. When staff members perceive real safety problems with appropriate and doable solutions backed by leadership supporting change, they may then adopt new behaviors to put the solution into practice. Initiatives to strengthen safety climate in nursing homes may thus be more likely to succeed and eventually increase resident safety if they first address their staff members’ readiness to change.

## Data Availability

The datasets generated and/or analysed during the current study are not publicly available due to VA data and privacy restrictions but are available from the corresponding author on reasonable request.

## Abbreviations

VA: Veterans Affairs
CLC: Community Living Centers
CESARS: CLC Employee Survey of Attitudes about Resident Safety
ORCA: Organizational Readiness to Change Assessment
ICC: Intraclass correlations
